# Bioconversion of Food and Green Waste into Valuable Compounds Using Solid-State Fermentation in Nonsterile Conditions

**DOI:** 10.3390/plants13243494

**Published:** 2024-12-13

**Authors:** Daniela Bulgari, Emanuela Gobbi, Paolo Cortesi, Gregorio Peron

**Affiliations:** 1 Department of Food Environmental and Nutritional Sciences, University of Milan, Via Celoria, 2, 20133 Milan, Italy; daniela.bulgari@unimi.it (D.B.); paolo.cortesi@unimi.it (P.C.); 2 Agri-Food and Environmental Microbiology Platform, Department of Molecular and Translational Medicine, University of Brescia, Viale Europa 11, 25123 Brescia, Italy; 3 Department of Molecular and Translational Medicine, University of Brescia, Viale Europa 11, 25123 Brescia, Italy; gregorio.peron@unibs.it

**Keywords:** *Trichoderma*, bioactive compounds, phenolic acid, biological nitrification inhibition, solid-state fermentation

## Abstract

Agro-industrial residues have transitions from being an environmental problem to being a cost-effective source of biopolymers and value-added chemicals. However, the efficient extraction of the desired products from these residues requires pretreatments. Fungal biorefinery is a fascinating approach for the biotransformation of raw materials into multiple products in a single batch. In this study, the ability of *Trichoderma asperellum* R to convert fruit scrap and green waste into value-added chemicals was tested in solid-state and in nonsterile conditions. A solid-state fermentation protocol for a tray bioreactor was developed using spawn as the inoculum for nonsterile substrates. *T. asperellum* R drove the fermentation of both substrates, shaping the metabolites that were enriched in the secondary plant metabolites. Strain R showed cellulase activity only when inoculated on fruit scraps, resulting in increased amounts of polysaccharides in the crude extract. This extract was also enriched in vanillic acid and limonoid, which are intriguing compounds due to the increasing interest in their potential as biological nitrification inhibitors or food additives. Finally, trimethoxybenzaldehyde, an interesting chemical building block, was identified in the extracts of the *Trichoderma*-guided fermentation. The overall results showed that the application of *T. asperellum* R has potential as a driver to facilitate the extraction of bioactive substances from nonsterile recalcitrant substrates.

## 1. Introduction

Food waste (FW) is the most common type of biowaste produced in the world. The quantity of the global FW created is related to food manufacturing, handling, storage, processing, distribution, and consumption [1]. The fruit and vegetable industry generates more waste than other food processing sectors, accounting for 25–30% of the total waste, which might include anything from peels and rinds to seeds, cores, rags, stones, pods, vines, skins, pomaces, and spoiled fruits and vegetables [2]. Along with FW, another type is the yard waste that is related to urbanization and to the increasing need for urban green spaces. Gardens and parks generate waste that consists of organic materials like grass clippings, hedge cuttings, pruning, leaves, and wood as well as inorganic materials like stones and soil [3]. The definition of green waste (GW) is not univocal in the literature, including garden waste, park waste, or the biodegradable fraction of municipal solid waste in this category. Herein, green waste is defined according to Langsdorf and colleagues as ‘grass and leaves collected from public parks, private gardens as well as cuttings from roadside greenery including a small amount of branches or other woody materials’ [4]. FW and GW valorization has received growing attention in terms of valuable compounds and bioenergy production. Different approaches have been developed from the more traditional ones, such as composting, vermicomposting, combustion, or incineration with appropriate landfill disposal, to more innovative ones. Modern techniques include the green extraction of valuable compounds, the chemical and fermentative conversion of waste into basic chemicals, as well as the manufacturing of functional materials like electrodes for electro-biotechnological applications through carbonization [4]. Solid-state fermentation (SSF) has been also successfully applied to pretreat lignocellulosic biomass to produce biofuels [5]. In SSF, the ability of fungi to release hydrolases is combined with other downstream metabolic pathways (fermentation) to directly convert lignocellulose into various metabolites [6]. Fungal pretreatment is defined as a biological process that uses rotting fungi to reduce the recalcitrance and enhance the enzymatic digestibility of lignocellulosic feedstock at low temperature, without added chemicals or wastewater generation [7]. White rot is the most well-known type of wood-rot fungi and is commonly used for pretreating the feedstock in biofuel production. Few studies have been carried out on soft-rot fungi such as *Trichoderma* spp. SSF has been successfully applied to convert FW into value-added products [1]. In previous works by our group, SSF with *Trichoderma* spp. strains has been revealed as a feasible strategy to convert agricultural digestate into profitable products with potential usefulness in the agrifood sector [8,9,10]. Specifically, SSF was shown to enrich the fermented substrate in citrate and chemical compounds with biostimulant activity such as gibberellins and to increase their extractability [8].

As a follow-up to our previous works, in this study, SSF was used to process GW (scrap from pruning) and FW (mix of fruits) with the aim of converting these materials into value-added products. Specifically, the main aims of this work were to (1) assess the suitability of unsterilized GW and FW to be used as substrates for medium-scale *T. asperellum* R fermentation; (2) monitor cellulase activity during fermentation; (3) assess metabolite changes during fermentation; (4) identify compounds with potential interest for the agrifood industry.

## 2. Results and Discussion

### 2.1. Trichoderma Growth on Different Waste-Based Substrates

**Figure 1:**
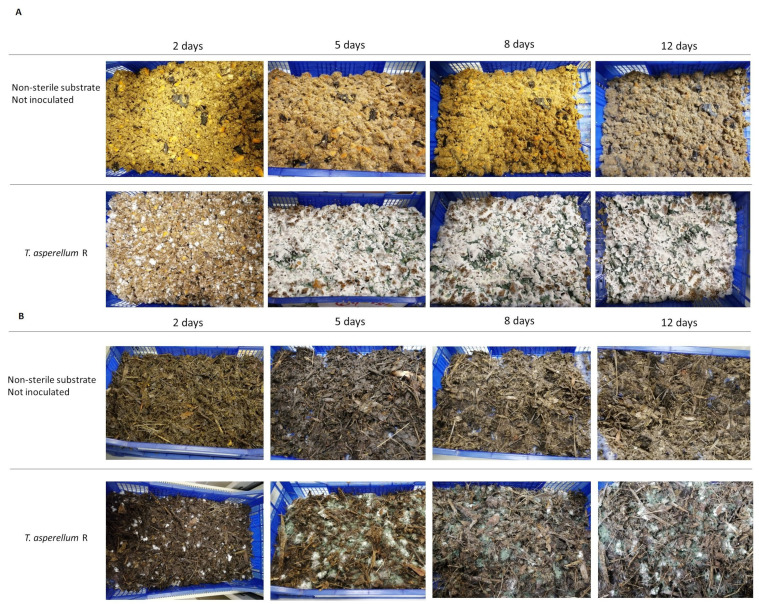
SSF of *Trichoderma asperellum* R after 2, 5, 8, 12 days of culture on (**A**) substrate 1 (mix of scrap fruit) and (**B**) substrate 2 (fruit mixed with GW).

Fungal bioconversion in the solid state is a fascinating approach for obtaining chemical compounds for pharmaceutical, biopesticide, and biofertilizers industries. Despite its potential, this approach has some drawbacks, including substrate sterilization requirement, a long pretreatment time, and the medium composition [11], which affect processing costs and ultimately impact profitability. A recent study highlighted that the use of a nonsterile substrate can effectively reduce the pretreatment cost at the biorefinery scale [5]. In the current work, SSF using a nonsterile substrate was conducted and an inoculation strategy, commonly used in fungal pretreatment, was applied to Ascomycota for the first time. *Trichoderma asperellum* R firstly grows on a sterilized substrate, and then the colonized substrate (spawn) is used as the inoculum for fungal SSF in nonsterile conditions. The use of an active growing fungus, instead of conidia, as the inoculum increases its probability of competing with the indigenous microflora and successfully colonizing the unsterilized substrate. Effectively, as shown in Figure 1, the inoculation of unsterilized substrate using the R strain spawn resulted in the prompt and successful colonization of both of the waste-based substrates inhabited by the indigenous microbial community. Fungal growth related to *Trichoderma* morphology was visible in both substrates inoculated with the R strain starting from 2 days after inoculation. Qualitative real-time PCR carried out on the total DNA extracted from the samples collected at different time points (2, 5, 8, 12 SSF days) confirmed the presence of *Trichoderma* sp. only in the inoculated substrates, proving that the *Trichoderma* spawn boosted the colonization of unsterilized substrate on the medium scale.

### 2.2. Cellulase Activity

Fungi belonging to the genus *Trichoderma* are versatile microorganisms that possess a complex enzymatic machinery including cellulases, hemicellulases, chitinases, proteases, and glucanases. *T. asperellum* R was previously characterized for cellulase and esterase activity during the SSF of a digestate-based substrate [10]. In the current work, cellulase activity was detected only in the crude extract collected from substrate 1 (scrap-fruit-based) fermented with *T. asperellum* R at 8 and 12 days of SSF (Table 1).

The highest cellulase activity (13.72 ± 2.6 mU/mL) was detected at 12 SSF days. No activity was found in either substrate 1 not inoculated with *T. asperellum* R or in the substrate 2. Despite the presence of cellulose in the substrates, the enzyme activity in the crude extract from substrate 2 could not be detected, possibly due to the presence of inhibitors. The fermentation process can generate undesirable molecules such as weak acids and phenols. These generated components can inhibit and/or deactivate cellulolytic enzyme activities and inhibit downstream microbial fermentation.

### 2.3. Crude Extract Metabolomics of Inoculated and Uninoculated Substrates

**Figure 2:**
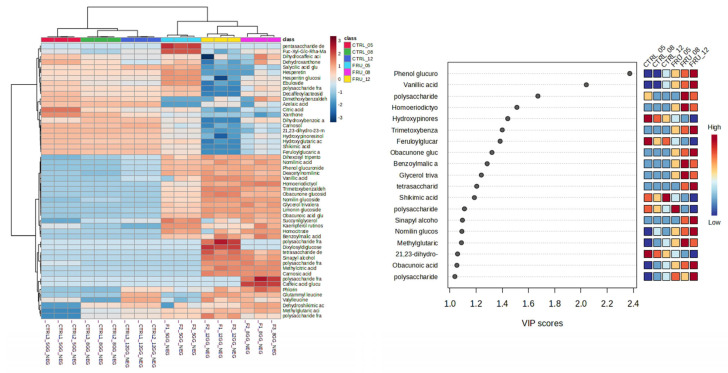
Heatmap (**left panel**) and VIP plot (**right panel**) obtained from untargeted analysis of crude extracts from fermented substrate 1 with *T. asperellum* R (FRU_05, FRU_08, FRU_12) and controls (CTRL_05, CTRL_08, CTRL_12). Extracts were obtained from substrates collected at 5, 8, and 12 days of SSF. Additional details on the metabolites reported in the VIP plot are included in Table 2.

**Figure 3:**
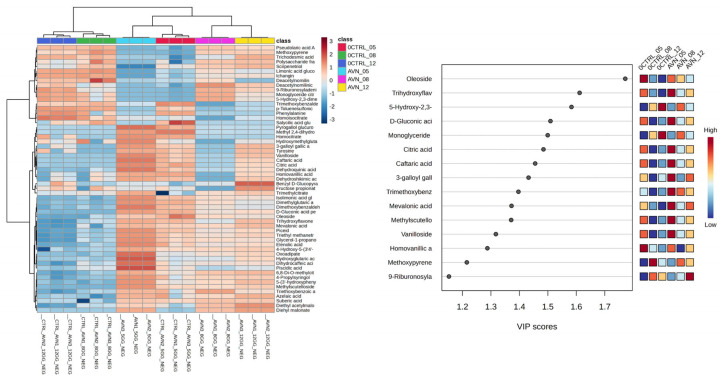
Heatmap (**left panel**) and VIP-plot (**right panel**) obtained from untargeted analysis of crude extracts from substrate 2 fermented with *T. asperellum* R (AVN_05, AVN_08, AVN_12) and controls (0CTRL_05, 0CTRL_08, 0CTRL_12). Extracts were obtained from substrate samples collected at 5, 8, and 12 days of SSF. Additional details on the metabolites reported in the VIP plot are included in Table 3.

An untargeted UPLC-MS-based analysis of the substrates allowed us to assess the chemical transformations that occurred spontaneously (control, uninoculated substrates) and during SSF with *T. asperellum* R (inoculated substrates). Two datasets were analyzed, i.e., one derived from the analysis of substrate 1 and the other derived from substrate 2. More than 100 variables were detected and included in the datasets: however, only a part of these (around 60) could be identified by means of MS data and hence were considered for multivariate analyses. The results of the two-dataset analysis are graphically reported in Figure 2 and Figure 3.

The heatmap in Figure 2 shows that the analyzed extracts were grouped in two clusters, i.e., uninoculated and inoculated. Subclusters were also observed in both groups, whose characteristics in the graph are related to the chemical variations induced by the time (days of fermentation). More importantly, the variables significantly describing the differences among samples were selected using a VIP plot, setting VIP > 1 as the threshold. They are listed in Table 2.

**Table 2:** Variables with highest discriminant power for the metabolic variations induced by SSF in substrate 1. Variables were selected using the VIP plot shown in Figure 2. Their identification was achieved considering the MS data reported in the table and the most probable molecular formula. Variables are listed from highest to lowest VIP value (see Figure 2).

RT (min)	*m*/*z*	Chemical Formula	Fragment	Tentative Identification	Chemical Class	Highest Yield (mg/100 g) *
5.7	269.0655	C_12_H_14_O_7_	93.0336	Phenol glucuronide	Polyphenol	1.80 ± 0.17 (t12)
5.7	167.0337	C_8_H_8_O_4_	123.0443	Vanillic acid	Polyphenol	0.45 ± 0.08 (t12)
5.1	1107.3456	C_40_H_68_O_35_	ND	Polysaccharide fragment 5	Carbohydrate	0.33 ± 0.03 (t8)
8.4	301.0706	C_16_H_14_O_6_	134.0353, 108.0199	Homoeriodictyol	Polyphenol	0.44 ± 0.03 (t8)
7.4	373.1287	C_20_H_22_O_7_	ND	Hydroxypinoresinol	Polyphenol	Decreased
6.3	195.0654	C_10_H_12_O_4_	163.0402, 119.0501	Trimetoxybenzaldehyde	Polyphenol	2.89 ± 0.15 (t12)
6	385.0758	C_16_H_18_O_11_	193.0523	Feruloylglucaric acid	Polyphenol	Decreased
7.6	633.2561	C_32_H_42_O_13_	471.2022	Obacunone glucoside	Limonoid	0.09 ± 0.01 (t12)
5.7	237.0394	C_11_H_10_O_6_	121.0299	Benzoylmalic acid	Polyphenol	0.21 ± 0.01 (t8)
7.2	343.2121	C_18_H_32_O_6_	ND	Glycerol trivalerate	Glyceride	0.08 ± 0.02 (t8)
2.1	603.1768	C_22_H_36_O_19_	ND	Tetrasaccharide derivative	Carbohydrate	0.18 ± 0.01 (t12)
2.4	173.0450	C_7_H_10_O_5_	93.1006	Shikimic acid	Polyphenol	Decreased
2.7	735.2189	C_27_H_44_O_23_	ND	Polysaccharide fragment 2	Carbohydrate	Decreased
6.9	209.0809	C_11_H_14_O_4_	181.0646, 153.0715	Sinapyl alcohol	Polyphenol	0.81 ± 0.04 (t12)
7.4	693.2758	C_34_H_46_O_15_	531.2235	Nomilin glucoside	Limonoid	1.12 ± 0.10 (t12)
2.5	161.0445	C_6_H_10_O_5_	ND	Methylglutaric acid	Organic acid	0.22 ± 0.01 (t8)
6.5	515.1912	C_27_H_32_O_10_	ND	21,23-Dihydro-23-methoxy-21-oxolimonin	Limonoid	Decreased
7.1	651.2651	C_32_H_44_O_14_	489.2133	Obacunoic acid glucoside	Limonoid	0.40 ± 0.05 (t12)
2.3	723.2194	C_26_H_44_O_23_	ND	Polysaccharide fragment 1	Carbohydrate	0.19 ± 0.01 (t12)

ND: not detected; * the column shows the highest yields of extraction expressed as mg/100 g of fresh substrate. The fermentation duration to achieve the highest yield is indicated in brackets (t8: 8 days of fermentation; t12: 12 days of fermentation). Decreased: concentration decreased compared to control (refer to Figure 2).

The two variables with the highest VIP value (>2) were those contributing the most to group differentiation. They were identified as phenol glucuronide and vanillic acid. Their amount gradually increased during SSF, reaching the highest value at 12 SSF days, while their amount did not change in the controls. This result suggests that the SSF of the inoculated substrates gradually released simple phenols, presumably from the degradation of the polyphenols in the fruits and the lignin in the sawdust and fruit peels [12]; these underwent phase-2 metabolization by *T. asperellum* R, i.e., glucuronidation. This activity was supported by the identification of vanillic acid and other two metabolites, namely, trimethoxybenzaldehyde and sinapyl alcohol, detected exclusively in the inoculated substrates. These compounds are catabolites formed by the microbial degradation of lignin, as already reported by other authors [13,14,15]. In particular, the formation of sinapyl alcohol and trimethoxybenzaldehyde occurs in microorganisms through the activity of the enzyme laccase [16], while vanillic acid is formed by the aerobic oxidation of vanillin [17]. This latter is of particular interest since it is widely used as a flavoring agent in the food, beverage, cosmetic, and pharmaceutical industries [18], and it is mainly obtained via chemical synthesis. Furthermore, vanillic acid is of interest to biofertilizer industries due as a biological nitrification inhibitor (BNI) [19]. BNIs are plant-derived compounds that have shown the ability to interfere with the oxidation of ammonia (NH_3_) to nitrite (NO_2_^−^) and subsequently to nitrate (NO_3_^−^) by microbial communities in the soil. The inhibition of the nitrification process reduces N losses through leaching, contributing to a more efficient fertilization strategy. Vanillic acid is reported to change soil microbial communities: it stimulates soil dehydrogenase activity and the soil’s microbial biomass carbon content, and it impacts soil bacteria and fungi community sizes and structures. Vanillic acid has also been reported to increase the relative abundances of taxa with denitrification capabilities while decreasing the relative abundance of those with nitrification capabilities [20].

Therefore, it is possible that VA and other phenolic acids influence plant growth through regulating rhizosphere nitrogen transformations, also affecting the rhizosphere soil microbial communities. Future studies will be carried out to investigate the activity of the *Trichoderma*-guided fermentation extract as a BNI.

Our results suggest that *T. asperellum* R, in association with other microorganisms recruited from the indigenous populations present on waste, contributes to degrade lignin through different mechanisms, which need to be further elucidated. Lignin is one of the most abundant organic materials in the world, along with cellulose [18], and it represents a relevant industrial waste [21]. The treatment of lignin-rich substrates with *T. asperellum* R in SSF conditions can represent a novel strategy for the disposal of this material and the sustainable production of valuable phenolic compounds such as vanillic acid, although further studies are needed to confirm this hypothesis.

Several other variables whose amounts increased with SSF were tentatively identified as oligo- and polysaccharides (Table 2). Also in this case, it was reasonable to consider that these compounds were formed as a result of the enzymatic degradation of the substrate by the fungus. Specifically, this may be related to the cellulolytic activity exerted by *T. asperellum* R, as previously reported. Interestingly, the amounts of several secondary plant metabolites increased in the substrates during SSF. Among these were the flavonoid homoeriodictyol and several limonoids characteristic of *Citrus* fruits such as obacunone glucoside, obacunoic acid, and nomilin glucoside (Table 2). These compounds have been already studied for their multiple bioactivities, ranging from antioxidant and anti-inflammatory [22] to antimicrobial and anticancer activities [23]. Hence, they may be of interest for use as nutraceuticals or drug candidates. Overall, the results of this study suggest that the SSF of fruit scraps with *T. asperellum* R may increase the extractability of bioactive secondary plant metabolites, presumably thanks to the degradation of cellulose fibers and lignin. These metabolites can be isolated from the substrate via a simple and sustainable aqueous extraction.

Regarding the SSF of substrate 2 (GW mixed with scrap fruits), the results were partially similar. As shown in the heatmap in Figure 3, in this case, the crude extract of the control and of the inoculated substrates were also different regarding their chemical composition, and the variations were induced by time and the SSF process. Notably, the largest difference was observed between the controls on days 8–12 and the *Trichoderma*-inoculated substrates collected at the same time points. The metabolites with the highest discriminant power for the different groups were highlighted using a VI -plot, as described above. They are listed in Table 3.

**Table 3:** Variables with highest discriminant power for the metabolic variations induced by SSF in substrate 2. Variables were selected using the VIP-plot shown in Figure 3. Their identification was achieved considering the MS data reported in the table and the most probable molecular formula. Variables are listed from highest to lowest VIP-value (see Figure 3).

RT (min)	*m*/*z*	Chemical Formula	Fragments	Tentative Identification	Chemical Class	Highest Yield (mg/100 g) *
5.4	389.1084	C_16_H_22_O_11_	227.0555	Oleoside	Iridoid	Decreased
6.4	593.1497	C_27_H_30_O_15_	473.1097	Trihydroxyflavone 6,8-diglucoside	Polyphenol	0.03 ± 0.00 (t5)
5.4	219.0671	C_12_H_11_O_4_	ND	5-Hydroxy-2,3-dimethyl-7-methoxychromone	Polyphenol	Decreased
4.1	405.1033	C_16_H_22_O_12_	ND	D-gluconic acid pentaacetate	Organic acid	0.01 ± 0.00 (t5)
4.3	255.0554	C_9_H_14_O_9_	129.0197	Monoglyceride citrate	Glyceride	Decreased
1.2	191.0187	C_6_H_8_O_7_	111.0012	Citric acid	Organic acid	0.48 ± 0.02 (t5)
5.4	311.0403	C_13_H_12_O_9_	135.0448	Caftaric acid	Polyphenol	0.29 ± 0.06 (t5)
4.9	323.0387	C_14_H_12_O_9_	169.0155	3-Galloyl gallic acid	Polyphenol	0.08 ± 0.01 (t5)
6.3	195.0956	C_10_H_12_O_4_	ND	Trimethoxybenzaldehyde	Polyphenol	0.49 ± 0.13 (t5)
3.0	147.0654	C_6_H_12_O_4_	ND	Mevalonic acid	Organic acid	0.08 ± 0.00 (t5)
4.4	393.1397	C_16_H_26_O_11_	231.0866	Methylscutelloside	Iridoid	0.02 ± 0.00 (t5)
7.1	313.0923	C_14_H_18_O_8_	151.0399	Vanilloside	Polyphenol	0.62 ± 0.11 (t5)
4.7	181.0501	C_9_H_10_O_4_	93.3195	Homovanillic acid	Polyphenol	Decreased
6.7	231.0861	C_17_H_12_O	ND	Methoxypyrene	Polycyclic aromatic hydrocarbon	Decreased
5.1	175.0238	C_6_H_8_O_6_	ND	9-Riburonosyladenine	Nucleoside	0.04 ± 0.01 (t12)

ND: not detected; * the column shows the highest yields of extraction expressed as mg/100 g of substrate. The time of fermentation to achieve the highest yield is indicated in brackets (t5: 5 days of fermentation; t12: 12 days of fermentation). Decreased: concentration decreased compared to control (refer to Figure 3).

The variable with the highest VIP value (>1.7) was identified as oleoside, a secoiridoid characteristic of olive plants, whose highest abundance was revealed in day 5 control substrates. The transformation induced by the growth of *T. asperellum* R increased the amounts of several secondary metabolites in the substrate on day 5 (Table 3), but, opposite to substrate 1, the amounts were generally lower on days 8 and 12 probably due to the progressive dehydration of the substrate. Among these compounds were several secondary plant metabolites such as the flavonoid trihydroxyflavone 6,8-diglucoside, the tannin 3-galloyl gallic acid, the hydroxycinnamate caftaric acid, the phenylaldehyde vanilloside, and the iridoid methylscutelloside. Some of these are of interest due to their bioactivity and their possible use in phytotherapy, as already reported by other authors (e.g., caftaric acid: [24]; methylscutelloside [25]). Trimethoxybenzaldehyde was found in the crude extracts of the *Trichoderma*-guided SSF of both substrates. Moreover, 2,4,5-trimethoxybenzaldehyde has been recently reported as a novel natural-product-inspired herbicide [26].

## 3. Materials and Methods

### 3.1. Trichoderma Isolate

*Trichoderma asperellum* strain R was previously characterized for its ability to produce enzymes via the solid-state fermentation of organic wastes [10]. The strain was originally isolated, identified, and conserved at the Mycothèque of Saida Messgo-Moumene, Laboratory of Research on Medicinal and Aromatic Plants, University of Blida 1, Algeria. Afterward, it was stored in the collection of the Agri-food and Environmental Microbiology Platform (PiMiAA), University of Brescia, Italy. *T. asperellum* R was grown on potato dextrose agar (PDA, Merck, Darmstadt, Germany) at 26 °C for 7 days and used for SSF substrate inoculation.

### 3.2. Spawn Preparation

*Trichoderma asperellum* R spawn was prepared using a protocol previously described [9]. Briefly, the SSF substrate was composed of a mix of apple, banana, grape, and kiwi not suitable for consumption (Table 4). Fruits, commonly present in the Italian supermarket, that were no longer fit for sale (food waste) were gathered from supermarket by the local nonprofit organization “Cauto”.

The substrate humidity was modified by adding carpentry sawdust (10% *w*/*w* mix fruits) to reach an SSF substrate humidity of 83.5%. The humidity was calculated according to IRSA-CNR Q 64/84. Briefly, moisture was calculated as the loss by weight of water by heating the waste to 105 ± 5 °C until a constant weight was reached. Two hundred and fifty grams of substrate was transferred in a plant micropropagation box (Micropoli, Cesano Boscone, Italy) and sterilized in an autoclave at 121 °C for 15 min for two consecutive cycles. The *T. asperellum* R conidia suspension (5 × 10^6^ conidia in sterile distilled water) was inoculated in each box. Control samples consisted of SSF substrates not inoculated with the fungus. All the substrates, four inoculated and two uninoculated, were incubated at 26°C and 40% RH under illumination of 12 h light/12 h dark cycles, using daylight tubes (24 W/m^2^, 9000 lx) in a climatic chamber (model 720, Binder GmbH, Tuttlingen, Germany) for 6 days.

### 3.3. Tray Bioreactor

The fungal strain was cultivated in plastic tray 60 cm long, 40 cm wide and 15 cm tall. We mixed 500 g of *T. asperellum* R spawn with 5400 g of substrate 1 and substrate 2 without previous sterilization (Table 5). Fruits in substrates 1 and 2 were provided by Cauto (Brescia, Italy) as mentioned above, while scrap from pruning was supplied by a local waste manager, which was collected during the cutting of roadsides, public parks, private gardens, and greenery, including a small amount of branches or other woody materials.

Substrate 2 was moistened with 2 L of sterilized water to reach a substrate humidity of 84%. Each substrate inoculated or not inoculated with *T. asperellum* R was distributed in two different trays and incubated in a fermentation cabinet in darkness for 12 days at 22 °C for 12 days. The SSF of both substrates was performed in duplicate.

### 3.4. Trichoderma asperellum R Detection

The *T. asperellum* R colonization of the nonsterile substrate was verified by qualitative real-time PCR. The total DNA from inoculated and uninoculated substrates was extracted using a previously developed method [9]. In detail, 50 g of inoculated and uninoculated substrates was sampled during fermentation (4, 8, and 12s day of fermentation) from the trays and ground with liquid nitrogen. Then, 500 mg of the obtained powder was used for nucleic acid extraction. The presence/absence of *T. asperellum* R in the inoculated and uninoculated substrate was determined using the oligonucleotide primers previously designed to specifically amplify *Trichoderma* calmodulin (TCal), as previously described [9,27].

### 3.5. Sampling and Crude Extract Preparation

We sampled 500 g of each substrate inoculated or not inoculated with *T. asperellum* R for aqueous extraction after 5, 8, and 12 days of fermentation. Then, 750 mL of sterilized deionized water was added to the substrate and mixed on an orbital shaker for 30 min at room temperature. The resulting suspension was filtered using Miracloth (Merck, Millipore, Darmstadt, Germany) and centrifuged at 10,000 rpm for 20 min. The supernatants were filtered through a 0.45 µm filter and stored at −20 °C until use. An aliquot of the crude extract (50 mL) was lyophilized and resuspended in 5 mL of sterile water for secondary metabolites analysis.

### 3.6. Cellulolytic Assay

The cellulase activity in the crude extracts was quantified using EnzChek™ cellulase substrate (Thermo Fisher Scientific, Waltham, MA, USA) following the manufacturer’s instructions. The quantification of cellulase (EC 3.2.1.4) was carried out using the standard curve method. Briefly, the crude extract was diluted (1:10) in a digestion buffer (100 mM sodium acetate buffer). A total of 50 µL of diluted sample and standard (cellulase from *Trichoderma reesei*, Merck, Sigma-Aldrich, Darmstadt, Germany) was added to 50 µL of substrate and incubated at room temperature for 30 min. The fluorescence was measured using a microplate reader (Ensight™, PerkinElmer, Waltham, MA, USA) with excitation/emission settings of 360/460 nm.

### 3.7. Metabolomic Analysis of Substrates

Extracts from fermented and uninoculated substrates were analyzed by UPLC-MS to assess their chemical composition. More specifically, an untargeted metabolomics approach was used to investigate the chemical variations induced over time and *Trichoderma*-guided fermentation of the substrates. Aqueous extracts were diluted 1:10 with MilliQ water, centrifuged at 13,300 rpm for 10 min, and directly used for analyses. These were performed on a Waters Acquity UPLC (Milford, MA, USA) coupled to a Waters Xevo G2 QToF-MS operating in negative mode (ESI-). A Waters Acquity UPLC BEH C18 column (2.1 × 50 mm, 1.7 μm) was used as the stationary phase and was maintained at 40 °C. The mobile phase was a mixture of 0.1% formic acid in water (A) and 0.1% formic acid in acetonitrile (B). The elution gradient was the following: 0–1 min, 98% A; 11 min, 15% A; 16 min, 0% A; 20 min, 0% A; 21 min, 98% A; 24 min, 98% A. The flow rate was set at 0.3 mL/min, and the injection volume was 1 μL. The mass range was 50–2000 Da. The MS parameters were as follows: sampling cone voltage, 40 V; source offset, 80 V; capillary voltage, 3.5 kV; nebulizer gas: N_2_ at a flow rate of 800 L/h; desolvation temperature, 450 °C. Mass accuracy and reproducibility were maintained by lock mass (leucine-enkephalin) infusion via lockspray at a flow rate of 20 μL/min. The MS^e^ experiment was performed with a fixed collision energy of 30 V to obtain structural information on the eluted compounds.

Chromatographic (retention time, RT; area under the curve, AUC, sum-normalized to total AUC) and MS (*m*/*z* values of parent ions) data of the eluted metabolites were extracted from the raw chromatograms and MS spectra using the MarkerLynx XS platform (Waters, Milford, MA, USA). The data matrix containing aligned and normalized data of around 100 features was exported as a .csv file and used to perform multivariate statistics in Metaboanalyst. Preliminary identification of variables (metabolites) was performed by comparing experimental *m*/*z* values, calculated molecular formulas, and fragmentation spectra with open-source databases such as Metlin (https://metlin.scripps.edu/landing_page.php?pgcontent=mainPage, accessed on 4 December 2024), KNApSAcK (http://www.knapsackfamily.com/KNApSAcK/, accessed on 4 December 2024), and PubChem (https://pubchem.ncbi.nlm.nih.gov/, accessed on 4 December 2024). In addition, the same data were compared with those in the available literature.

The semiquantification of the variables associated to the shifting of chemical composition in fermented substrates was performed by using calibration curves of chlorogenic acid for phenolic acids (concentration range: 93–9300 ppb), rutin for flavonoids (concentration range: 8–8000 ppb), azadirachtin for terpenoids (concentration range: 50–5000 ppb), citric acid for organic acids (concentration range: 5–5000 ppb), and glucose for carbohydrates (concentration range: 10–10,000 ppb). The results are reported as concentration of analyte in fresh substrate (mg/100 g) and are expressed as mean ± standard deviation of three independent measurements.

### 3.8. Statistical Analyses

Multivariate analysis of metabolomics data was performed in Metaboanalyst v.6.0 (https://www.metaboanalyst.ca). Data were sum-normalized, log-transformed, and Pareto-scaled before analysis. Clustering analysis was performed by a heatmap, where the abundancy of the metabolites in the different samples was represented, as well as the clustering of the samples. The selection of the most important variables (metabolites) associated to the differences among clusters was performed using a variable importance in projection (VIP) plot, obtained through a partial least squares discriminant analysis (PLS-DA). Variables with VIP > 1 are highlighted, and their significance (*p* < 0.05) was assessed through one-way ANOVA.

## 4. Conclusions

Overall, these results indicate that the fermentation of recalcitrant organic residues such as FW and GW via the SSF with *T. asperellum* R may be a sustainable pretreatment to increase the amounts of extractable bioactive compounds from low-value materials such as grass clippings and fruit waste.

The fungal SSF of waste in nonsterile condition should be further investigated to develop new products with soil nitrification-inhibiting features to improve agricultural productivity and environmental sustainability. As nitrogen is one of the more critical factors in agricultural ecosystems for both yield and environmental issues, the enrichment of the crude extracts in secondary plant metabolites (phenolic alcohol and flavonoid), which could affect plant growth by means of regulating rhizosphere nitrogen transformations, could be pivotal.

## Figures and Tables

**Table 1:** Cellulase activity detected in the crude extract collected on different days of fermentation in inoculated and uninoculated substrates.

		Cellulase (mU/mL)	
Substrate	SSF Days	Control	*T. asperellum*
1	5	-	-
1	8	-	1.52 ± 0.01
1	12	-	13.72 ± 2.6

**Table 4:** Constituents of the culture substrate for spawn preparation of *Trichoderma asperellum* R.

Component	% (*w*/*w*)	Amount per Culture (g)
Table grape	25	62.5
Apple	25	62.5
Banana	25	62.5
Kiwi	25	62.5
**Modifier**		
Wood sawdust	~10 of total weight	

**Table 5:** Constituents of the culture substrates for solid-state fermentation of *Trichoderma asperellum* R in tray bioreactor.

Component	% (*w*/*w*)	Amount per Culture (g)
	**Substrate 1**	
Orange	25	1350
Apple	25	1350
Banana	25	1350
Kiwi	25	1350
**Modifier**		
Wood sawdust	~10 of total weight	
	**Substrate 2**	
Scrap from pruning	70	3780
Substrate 1	30	1620

## Data Availability

The original contributions presented in this study are included in this article/Supplementary Materials; further inquiries can be directed to the corresponding author.

## Supplementary Materials

The following supporting information can be downloaded at: https://www.mdpi.com/article/10.3390/plants13243494/s1, Table S1: Identified metabolites that were included in the dataset for the metabolomics analysis of substrate 1. Variables are listed in order of retention time (RT). Highlighted variables are those with the highest VIP and are included in Table 2; Table S2: Identified metabolites that were included in the dataset for the metabolomics analysis of substrate 2. Variables are listed in order of retention time (RT). Highlighted variables are those with the highest VIP and are included in Table 3.
