# Supplementary material for: Bioconversion of Food and Green Waste into Valuable Compounds Using Solid-State Fermentation in Nonsterile Conditions
Source: Plants (Basel). 2024 Dec 13;13(24):3494. doi: 10.3390/plants13243494 (PMC11728819; doi:10.3390/plants13243494)
Supplement: Supplementary file 1 [file plants-13-03494-s001.zip › plants-3241156-supplementary.pdf]

Bioconversion of agro-industrial waste in solid-state nonsterile condition: metabolomic assessment and identification of valuable compounds

Daniela Bulgari[1], Emanuela Gobbi[2], Gregorio Peron[3]

[1]Department of Food Environmental and Nutritional Sciences, University of Milan, via Celoria, 2, 20133, Milan, Italy

[2]Agri-Food and Environmental Microbiology Platform, Department of Molecular and Translational Medicine, University of Brescia, viale Europa, 11, 25123 Brescia, Italy

[3]Department of Molecular and Translational Medicine, University of Brescia, viale Europa, 11, 25123, Brescia, Italy

Table S1. Identified metabolites that were included in the dataset for the metabolomics analysis of substrate 1. Variables are listed in order of retention time (RT). Highlighted variables are those with the highest VIP and were included in Table 4.

**RT (min)**	**m/z**	**Chemical formula**	**Fragments**	**Tentative identification**	**Chemical class**
1.3	191.0187	C6H8O7	111.0013	Citric acid	Organic acid
2	147.0288	C5H8O5	129.0189	Hydroxyglutaric acid	Organic acid
2.1	603.1768	C22H36O19	ND	tetrasaccharide derivative	Carbohydrate
2.3	723.2194	C26H44O23	ND	polysaccharide fragment 1	Carbohydrate
2.4	205.0342	C7H10O7	161.0441	Homocitrate	Organic acid
2.4	169.0132	C7H10O5	93.1006	Shikimic acid	Polyphenol
2.5	161.0445	C6H10O5	ND	Methylglutaric acid	Organic acid
2.7	735.2189	C27H44O23	ND	polysaccharide fragment 2	Carbohydrate
3.1	677.2139	C25H42O21	ND	pentasaccharide derivative	Carbohydrate
3.2	605.1927	C22H38O19	ND	Dixylosyldiglucose	Carbohydrate
3.2	259.1287	C11H20N2O 5	ND	Glutammyl leucine	Peptide
3.5	737.2347	C27H46O23	ND	Dihexosyl tripentoside	Carbohydrate
3.5	205.0342	C7H10O7	191.0189, 111.0014	Methylcitric acid	Organic acid
3.8	839.2661	C31H52O26	689.2142	pentaarabinoside glucoside	Carbohydrate

3.8	265.0914	C10H18O8	ND	Succynilglycerol	Glyceride
4.1	171.0283	C7H8O5	127.0403	Dehydroshikimic acid	Polyol
4.1	1311.4105	C48H80O41	ND	Man-Glc-Gal-Rha-Man-Glc-Gal-Glc	Carbohydrate
4.5	645.1871	C24H38O20	ND	polysaccharide fragment 3	Carbohydrate
4.5	813.2505	C29H50O26	ND	polysaccharide fragment 4	Carbohydrate
4.8	193.0279	C13H5O2	ND	Dehydroxanthone	Polyphenol
4.9	153.0181	C7H6O4	135.0076, 109.0299	Dihydroxybenzoic acid	Polyphenol
4.9	195.0488	C13H8O2	ND	Xanthone	Polyphenol
5	779.2454	C29H48O24	617.1937	Fuc-Xyl-Glc-Rha-Man	Carbohydrate
5.1	1107.3456	C40H68O35	ND	polysaccharide fragment 5	Carbohydrate
5.3	1457.4676	C54H90O45	ND	polysaccharide fragment 6	Carbohydrate
5.5	355.0656	C15H16O10	179.0344	Caffeic acid glucuronide	Polyphenol
5.5	313.055	C13H14O9	137.0232	Salycilic acid glucuronide	Polyphenol
5.6	329.1745	C20H26O4	ND	Carnosol	Terpene
5.7	237.0394	C11H10O6	121.0299	Benzoylmalic acid	Polyphenol
5.7	181.0493	C9H10O4	163.0401	Dihydrocaffeic acid	Polyphenol
5.7	269.0655	C12H14O7	93.0336	Phenol glucuronide	Polyphenol
5.7	167.0337	C8H8O4	123.0443	Vanillic acid	Polyphenol
5.9	443.1906	C21H32O10	ND	Ebuloside	Iridoid
5.9	229.1545	C11H22N2O3	ND	Valylleucine	Peptide
6	385.0758	C16H18O11	193.0523	Feruloylglucaric acid	Polyphenol
6.2	593.1506	C27H30O15	285.0402	Kaempferol rutinoside	Polyphenol
6.3	195.0654	C10H12O4	163.0402, 119.0501	Trimetoxybenzaldehyde	Polyphenol
6.5	515.1912	C27H32O10	ND	21,23-dihydro-23-methoxy-21-oxolimonin	Limonoid
6.6	461.1651	C20H30O12	ND	Decaffeoylacteoside	Polyphenol
6.7	651.2661	C32H43O14	489.2141	Deacetylnomilinic acid glucoside	Limonoid
6.8	165.0547	C9H10O3	137.0246	Dimethoxybenzaldehyde	Polyphenol
6.9	649.2496	C32H42O14	487.1972	Limonin glucoside	Limonoid
6.9	209.0809	C11H14O4	181.0646, 153.0715	Sinapyl alcohol	Polyphenol
7.1	651.2651	C32H44O14	489.2133	Obacunoic acid glucoside	Limonoid
7.1	287.076	C12H16O8	125.0244	Phlorin	Polyphenol
7.2	343.2121	C18H32O6	ND	Glycerol trivalerate	Glyceride
7.3	187.097	C9H16O4	ND	Azelaic acid	Organic acid
7.3	301.0712	C16H14O6	164.0124, 151.0039	Hesperetin	Polyphenol
7.6	463.1235	C22H24O11	301.0718	Hesperitin glucoside	Polyphenol
7.4	373.1287	C20H22O7	ND	Hydroxypinoresinol	Polyphenol

7.4	693.2758	C34H46O15	531.2235	Nomilin glucoside	Limonoid
7.4	711.2869	C34H48O16	ND	Nomilinic acid	Limonoid
7.6	633.2561	C32H42O13	471.2022	Obacunone glucoside	Limonoid
8.4	301.0706	C16H14O6	134.0353, 108.0199	Homoeriodictyol	Polyphenol
8.9	331.1902	C20H28O4	288.0012	Carnosic acid	Terpene
1.3	191.0187	C6H8O7	111.0013	Citric acid	Organic acid

Table S2. Identified metabolites that were included in the dataset for the metabolomics analysis of substrate 2. Variables are listed in order of retention time (RT). Highlighted variables are those with the highest VIP and were included in Table 5.

**RT (min)**	**m/z**	**Chemical formula**	**Fragments**	**Tentative identification**	**Chemical class**
1.2	191.0187	C6H8O7	111.0012	Citric acid	Organic acid
2	157.0137	C6H6O5	ND	Oxoadipate	Organic acid
2.2	147.0287	C5H8O5	ND	Hydroxyglutaric acid	Organic acid
2.3	205.034	C7H10O7	161.0447	Homocitrate	Organic acid
2.5	161.0452	C6H10O5	ND	Hydroxymethylglutaric acid	Organic acid
2.5	233.0661	C9H14O7	191.0183	Trimethylcitrate	Organic acid
2.7	205.0337	C7H10O7	161.0444	Homoisocitrate	Organic acid
3	147.0654	C6H12O4	ND	Mevalonic acid	Organic acid
3.1	231.0865	C10H16O6	ND	Glycerol-1-propanoate diacetate	Polyol
3.2	159.0652	C7H12O4	113.1045, 87.0446	Diethyl malonate	Organic acid
3.9	189.0394	C7H10O6	ND	Dehydroquinic acid	Polyol
4	171.029	C7H8O5	127.0403	Dehydroshikimic acid	Polyol
4	251.0765	C9H16O8	ND	Fructose propionate	Carbohydrate
4.1	405.1033	C16H22O12	ND	D-Gluconic acid pentaacetate	Organic acid
4.3	255.0554	C9H14O9	129.0197	Monoglyceride citrate	Glyceride
4.3	173.0808	C8H14O4	ND	Suberic acid	Organic acid
4.4	393.1397	C16H26O11	231.0866	Methylscutelloside	Iridoid
4.6	401.1077	C17H22O11	339.1085, 225.0775	4-Hydroxy-5-(3'4'-dihydroxyphenyl)-valeric acid-O-glucuronide	Polyphenol
4.7	181.0501	C9H10O4	93.3195	Homovanillic acid	Polyphenol
4.7	167.0348	C8H8O4	ND	Methyl 2,4-dihydroxyphenylacetate	Polyphenol
4.9	164.0712	C9H11NO2	ND	Phenylalanine	Amino acid
4.9	323.0387	C14H12O9	169.0155	3-Galloyl gallic acid	Polyphenol
5.1	175.0238	C6H8O6	ND	9-Riburonosyladenine	Nucleoside
5.1	231.0869	C10H16O6	ND	Triethoxybenzoic acid	Polyphenol

5.2	301.056	C12H14O9	125.0237	Pyrogallol glucuronide	Polyphenol
5.4	219.0671	C12H11O4	ND	5-Hydroxy-2,3-dimethyl-7-methoxychromone	Polyphenol
5.4	311.0403	C13H12O9	135.0448	Caftaric acid	Polyphenol
5.4	389.1084	C16H22O11	227.0555	Oleoside	Iridoid
5.5	187.097	C9H16O4	ND	Azelaic acid	Organic acid
5.5	201.0763	C9H14O5	ND	Diethyl acetylmalonate	Organic acid
5.5	313.0561	C13H14O9	137.0246	Salycilic acid glucuronide	Polyphenol
5.7	180.0661	C9H11NO3	ND	Tyrosine	Amino acid
5.8	401.1084	C17H22O11	225.0766	5-(3'-hydroxyphenyl)-gamma-hydroxyvaleric acid -4'-O-glucuronide	Polyphenol
5.8	241.0712	C11H14O6	209.0459	Elenolic acid	Iridoid
5.8	171.0116	C7H8O3S	79.9584	p-Toluenesulfonic acid	Benzenesulfonate
6	255.0505	C11H12O7	211.0601	Piscidic acid	Polyphenol
6.3	195.065	C10H12O4	ND	Triethyl methanetricarboxylate	Organic acid
6.3	195.0956	C10H12O4	ND	Trimethoxybenzaldehyde	Polyphenol
6.4	593.1497	C27H30O15	473.1097	Trihydroxyflavone 6,8-diglucoside	Polyphenol
6.5	269.1018	C13H18O6	107.0511	Benzyl D-Glucopyranoside	Polyphenol
6.5	159.0652	C7H12O4	ND	Dimethylglutaric acid	Organic acid
6.5	667.2612	C32H44O15	505.2076	Isolimonic acid glucoside	Limonoid
6.6	357.0817	C15H18O10	223.0608	Dihydrocaffeic acid 3-O-glucuronide	Polyphenol
6.7	327.0834	C18H16O6	ND	6,8-Di-O-methylcitreoisocoumarin	Polyphenol
6.7	231.0861	C17H12O	ND	Methoxypyrene	Polycyclic aromatic hydrocarbon
6.7	215.0919	C10H16O5	ND	Trichodesmic acid	Terpene
6.8	389.1227	C20H22O8	ND	Piceid	Polyphenol
6.9	165.055	C9H10O3	149.0249	Dimethoxybenzaldehyde	Polyphenol
6.9	505.2082	C26H34O10	505.2071	Limonic acid glucoside	Limonoid
7.1	313.0923	C14H18O8	151.0399	Vanilloside	Polyphenol
7.4	489.1456	C17H30O16	ND	Polysaccharide fragment	Carbohydrate
7.5	489.2125	C26H34O9	ND	Deacetylnomilinic acid	Limonoid
7.8	549.2341	C28H38O11	387.1804	Pseudolaric acid A O-beta-D-glucopyranoside	Terpene
8.1	195.1016	C11H16O3	180.0799, 165.0551	4-Propylsyringol	Polyphenol
8.2	471.2016	C26H32O8	ND	Deacetylnomilin	Limonoid
8.6	487.1962	C26H32O9	ND	Ichangin	Limonoid
